# Supplementary material for: The development of synaptic transmission is time-locked to early social behaviors in rats
Source: Nat Commun. 2019 Mar 13;10:1195. doi: 10.1038/s41467-019-09156-3 (PMC6416358; doi:10.1038/s41467-019-09156-3)
Supplement: Supplementary file 1 — Supplementary Information [file 41467_2019_9156_MOESM1_ESM.pdf]

Supplementary Information

The development of synaptic transmission is time-locked to early social behaviors in rats.

Naskar et al.

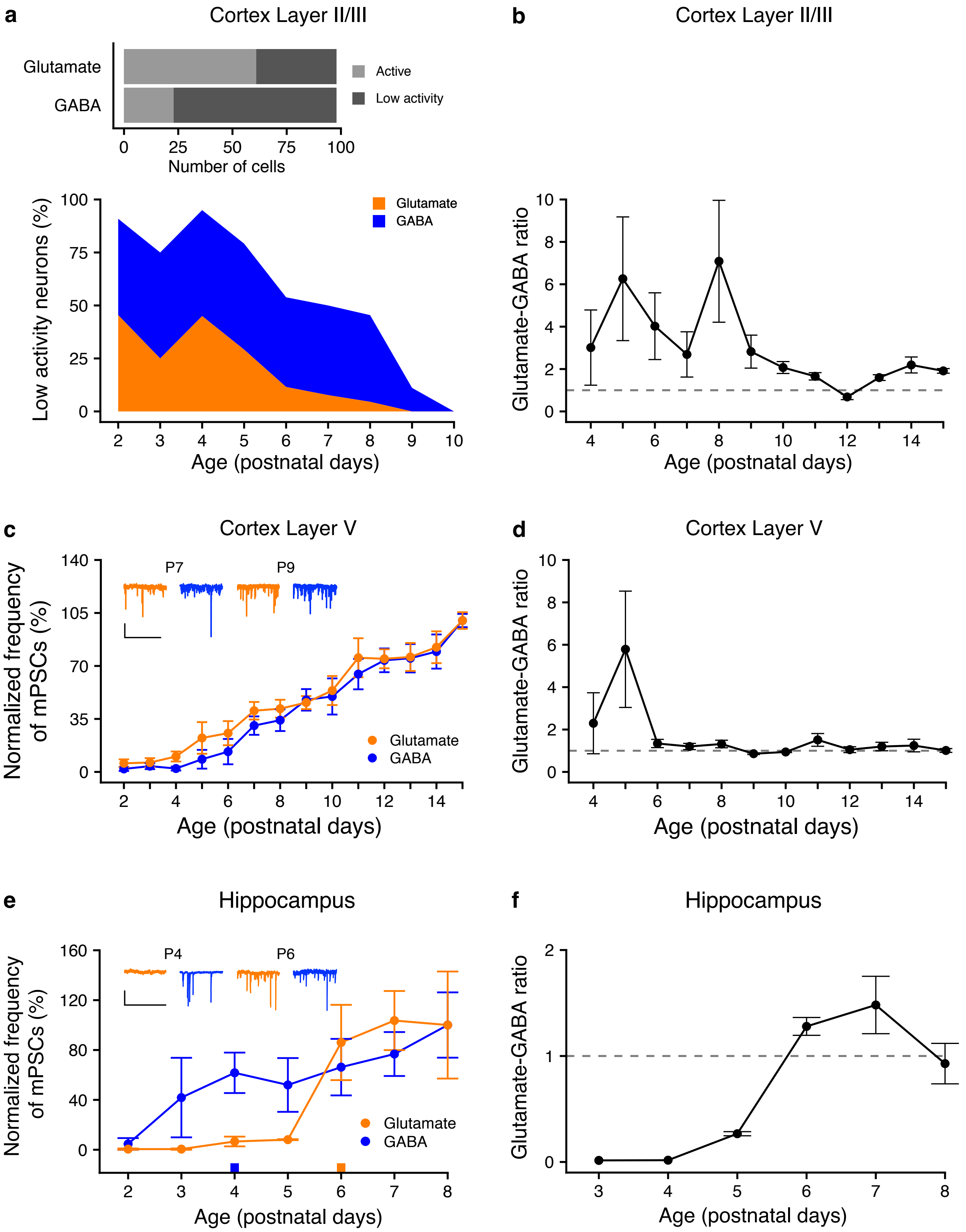
a
Cortex Layer II/III
Glutamate
GABA
Active
Low activity
0
25
50
75
100
Number of cells
Low activity neurons (%)
Glutamate
GABA
Age (postnatal days)
b
Cortex Layer II/III
Glutamate-GABA ratio
Age (postnatal days)
c
Cortex Layer V
P7
P9
Normalized frequency of mPSCs (%)
Glutamate
GABA
Age (postnatal days)
d
Cortex Layer V
Glutamate-GABA ratio
Age (postnatal days)
e
Hippocampus
P4
P6
Normalized frequency of mPSCs (%)
Glutamate
GABA
Age (postnatal days)
f
Hippocampus
Glutamate-GABA ratio
Age (postnatal days)

5

Supplementary Figure 1. Layers II/III and V of somatosensory cortex and the hippocampus show a different sequence of functional synaptogenesis. **a** Top: Horizontal bar plot showing a smaller number of ‘low activity’ cells (dark gray, see methods) among neurons with glutamatergic vs GABAergic currents for the total of all neurons recorded between P2 and P10 in Fig. 1b ($\chi^2_{(1)} = 30$, $P<0.001$; Pearson’s Chi-squared test, $N = 98$). Bottom: Quantification of the percentage of low activity cells amongst glutamatergic (orange) and GABAergic (blue) neurons recorded for each day from P2 to P10. **b** Ratio of glutamate-GABA frequency of mPSCs ± SEM from normalized data in Fig. 1b remains above 1 up to P11, indicating predominance of glutamatergic currents. **c** Development of glutamatergic and GABAergic mPSCs ± SEM from somatosensory cortex neurons of infra-granular layers; $N = 116$ cells in total, from 27 animals (4-10 neurons per postnatal age). Values are normalized on the mPSC means at P15. Inset: example traces of glutamatergic and GABAergic mPSCs. Scale bars: 20 pA, 2 s. **d** Ratio of glutamate-GABA frequency of mPSCs ± SEM from normalized data in (**c**) remains mostly close to 1 throughout the first two postnatal weeks, indicating no strong prevalence of either glutamatergic or GABAergic currents in infragranular layers. **e** Development of glutamatergic and GABAergic mPSCs ± SEM in CA1 hippocampal neurons; $N = 31$ neurons total, from 9 animals (2-6 neurons per postnatal age). Values are normalized on the mPSC means at P8. Squares at the bottom indicate the first postnatal days where a mPSC frequency significantly larger than 0 Hz was identified (one-tailed one-sample Wilcoxon signed rank test against a theoretical value of 0, W=21, $P<0.05$). Inset: example traces of glutamatergic and GABAergic mPSCs. Scale bars: 20 pA, 2 s. **f** Ratio of glutamate-GABA frequency of mPSCs ± SEM from normalized data in (**e**) remains below 1 from P3 to P5, indicating predominance of GABAergic currents in early hippocampal development.

Low activity cells were excluded from the analysis in (**b, d, f**) and dotted line indicates a ratio of 1 for reference.

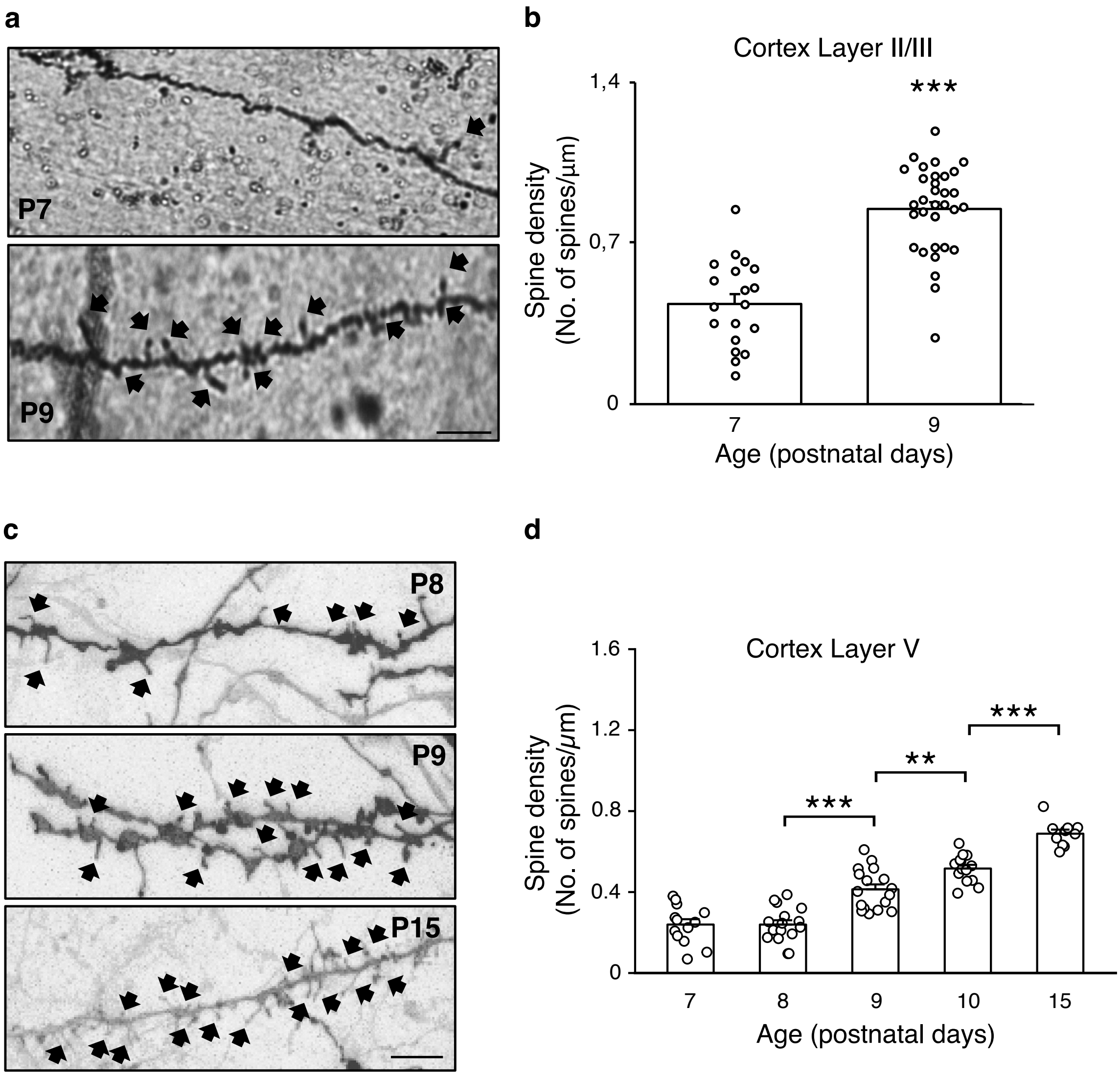

Supplementary Figure 2. Layer II/III show higher spine density at P9 vs P7 and Layer V shows a progressive spine density increase during development. **a** Representative images of secondary dendrites of Layer II/III neurons processed for Golgi staining. Black arrows point to spine-like protrusions. Scale bar: 8 µm. **b** Quantification of average spine density ± SEM and single dendritic branch data points of Layer II/III neurons (6 animals total; two-sample *t*-test, t=-7.5, degrees of freedom=38). **c** Confocal images of secondary dendrites of Layer V neurons stained with DiI. Black arrows indicate spine-like protrusions. Scale bar: 8 µm. **d** Average spine density ± SEM and single dendritic branch data points of Layer V neurons at different postnatal days (6 animals total; one-way ANOVA, $F_{(4,65)}$=60.5, *P*<0.001; pairwise *t*-test comparisons with Holm's correction). ***P*<0.01, ****P*<0.001.

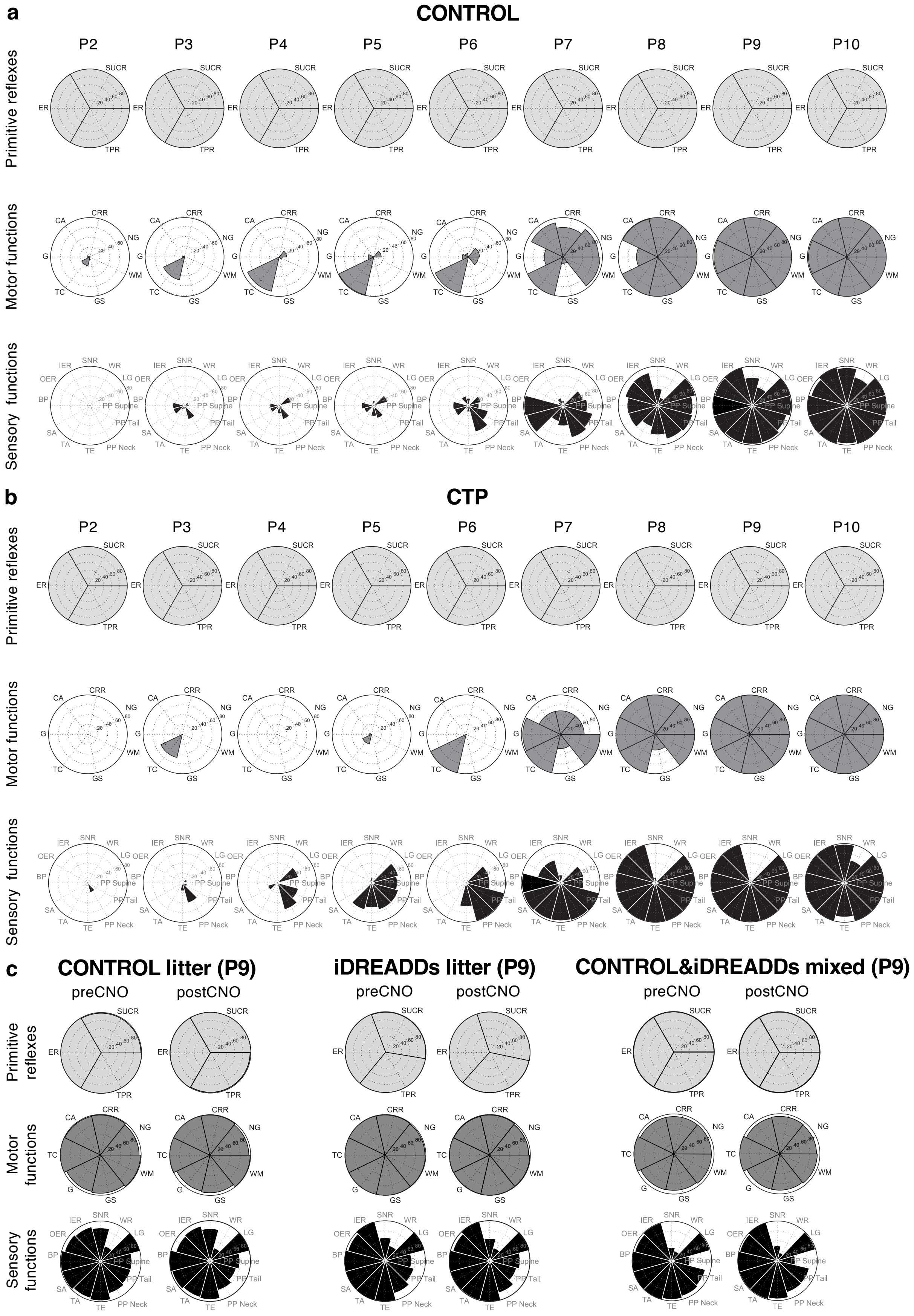
a
CONTROL
P2
P3
P4
P5
P6
P7
P8
P9
P10
Primitive reflexes
SUCR
ER
TPR
20
40
60
80
Motor functions
CA
CRR
NG
G
WM
TC
GS
Sensory functions
IER
SNR
WR
LG
OER
BP
PP Supine
PP Tail
SA
TA
TE
PP Neck
b
CTP
c
CONTROL litter (P9)
iDREADDs litter (P9)
CONTROL&iDREADDs mixed (P9)
preCNO
postCNO

Supplementary Figure 3. Sensory-motor functions develop by P7 in Control and CTP-treated animals and are independent of Layer II/III neurotransmission. **a**, **b** Polar plots of raw data of primitive reflexes (light gray), motor (dark gray) and sensory functions (black) in Controls and CTP-treated animals. Data were collected in the same experiments described in Fig. 2b and 4e-f. **c** Polar plots of P9 litters electroporated with different vector configurations before and after CNO administration. Left: Control litters electroporated with GFP (*N* = 9, total pups = 90); middle: litters electroporated with iDREADDs in all pups (*N* = 11, total pups = 110); right: litters electroporated in half of the pups with control GFP vector and in half of the pups with iDREADDs (*N* = 11, total pups = 110). Data were collected in the experiments described in Fig. 3f, h and l. For a description of the acronyms of the scored behaviors see Supplementary Table 1.

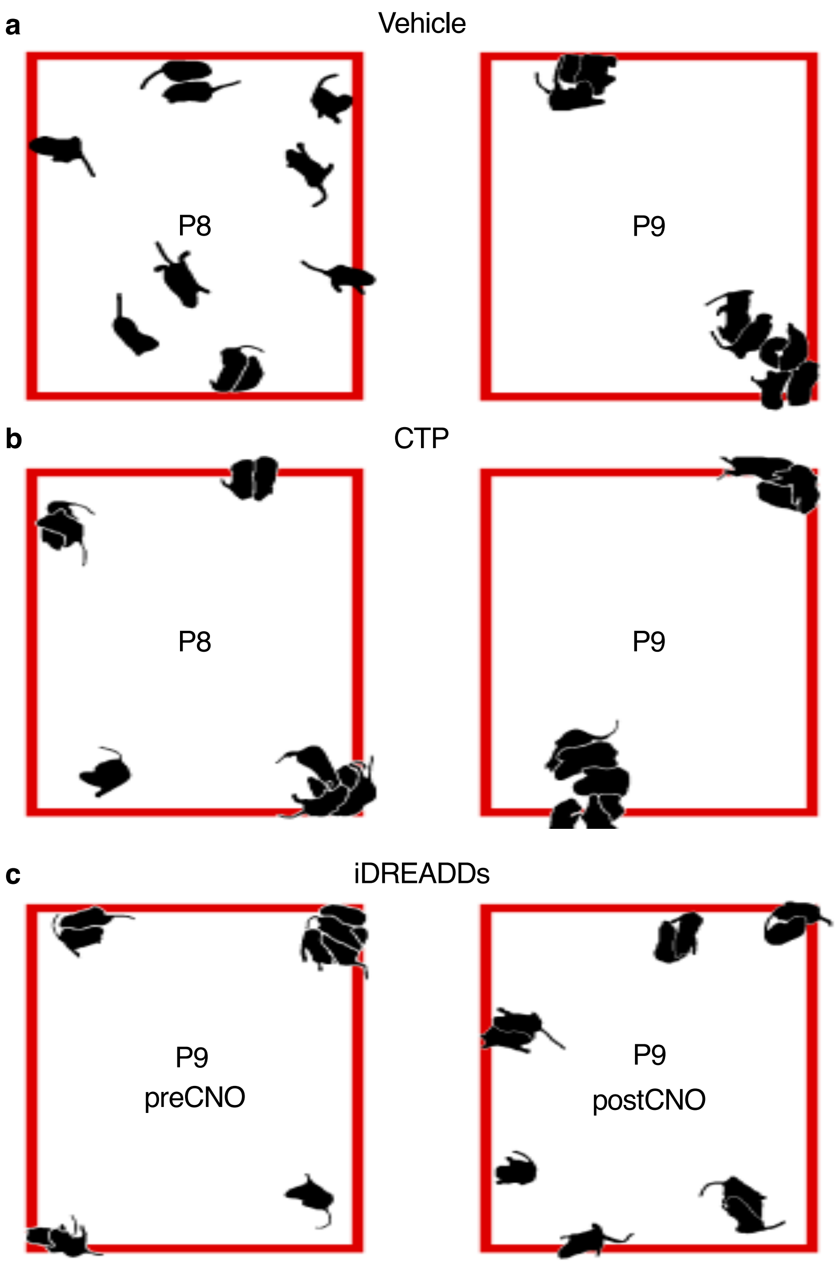

Supplementary Figure 4. Full huddling behavior develops at P9, it is anticipated by CTP treatment and depends on somatosensory cortex activity. **a**, **b** Silhouettes of last-frame snapshots of typical experiments with animals treated with vehicle or CTP. **c** Silhouettes of last-frame snapshots of typical experiments with animals transfected with iDREADDs before and after CNO administration.

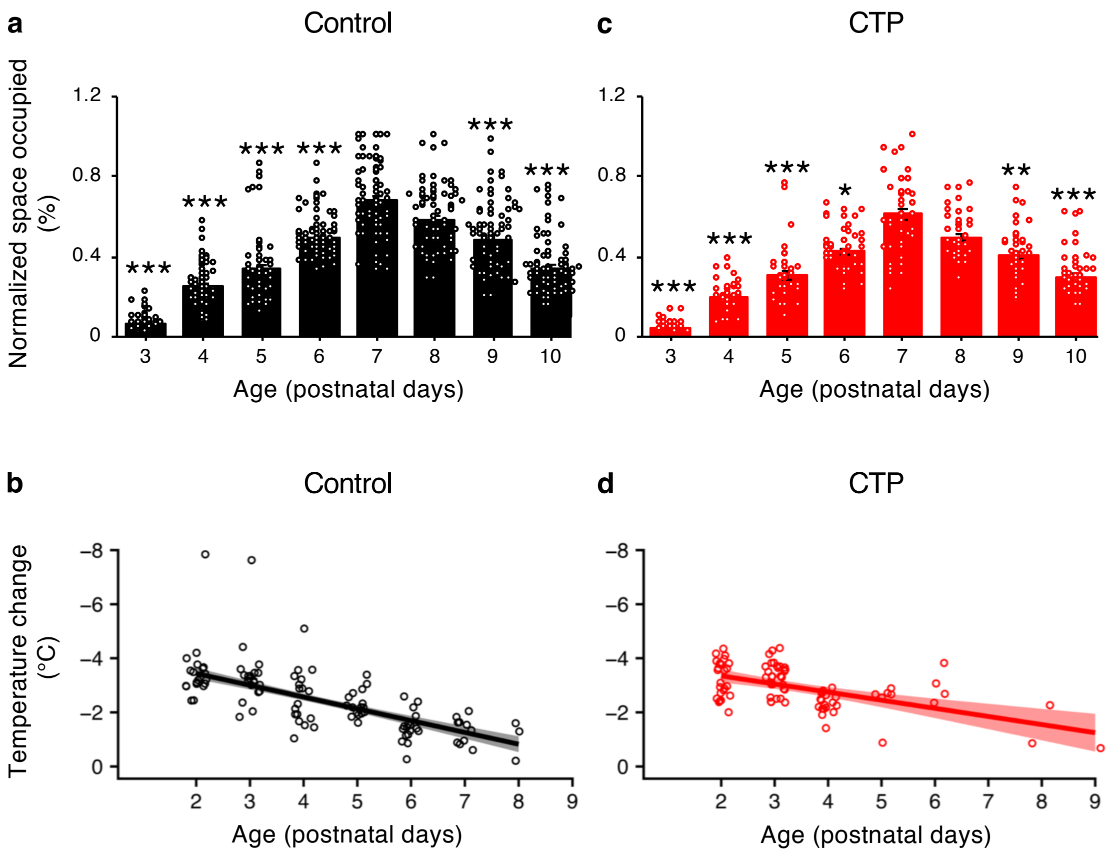

Supplementary Figure 5. The developmental time course of motor ability and temperature regulation does not mimic the huddling one. **a**, **c** Average ± SEM and single animal data of the normalized total space occupied in the arena by animals during the experiments described in Fig. 4g. Raw values of single animals from each litter were normalized to the value of the animal that occupied the maximum space for the same litter at any age. In both Controls (black) and CTP-treated animals (red), the space occupied peaked at P7; Kruskal-Wallis test $\chi^2_{(7)}$=420, P<0.001 and $\chi^2_{(7)}$=270, P<0.001 respectively; Dunn's *post hoc* test against P7 with Holm's correction (**P*<0.05, ***P*<0.01, ****P*<0.001). **b**, **d** Drop in body temperature in animals that never huddled at any given age in the experiments described in Fig. 4g. Graphs show a negative correlation between the drop of body temperature and the increase in age of Control (r=0.68, *P*<0.001, Pearson Product-Moment Correlation) and CTP-treated (r=0.55, *P*<0.001) non-huddling animals.

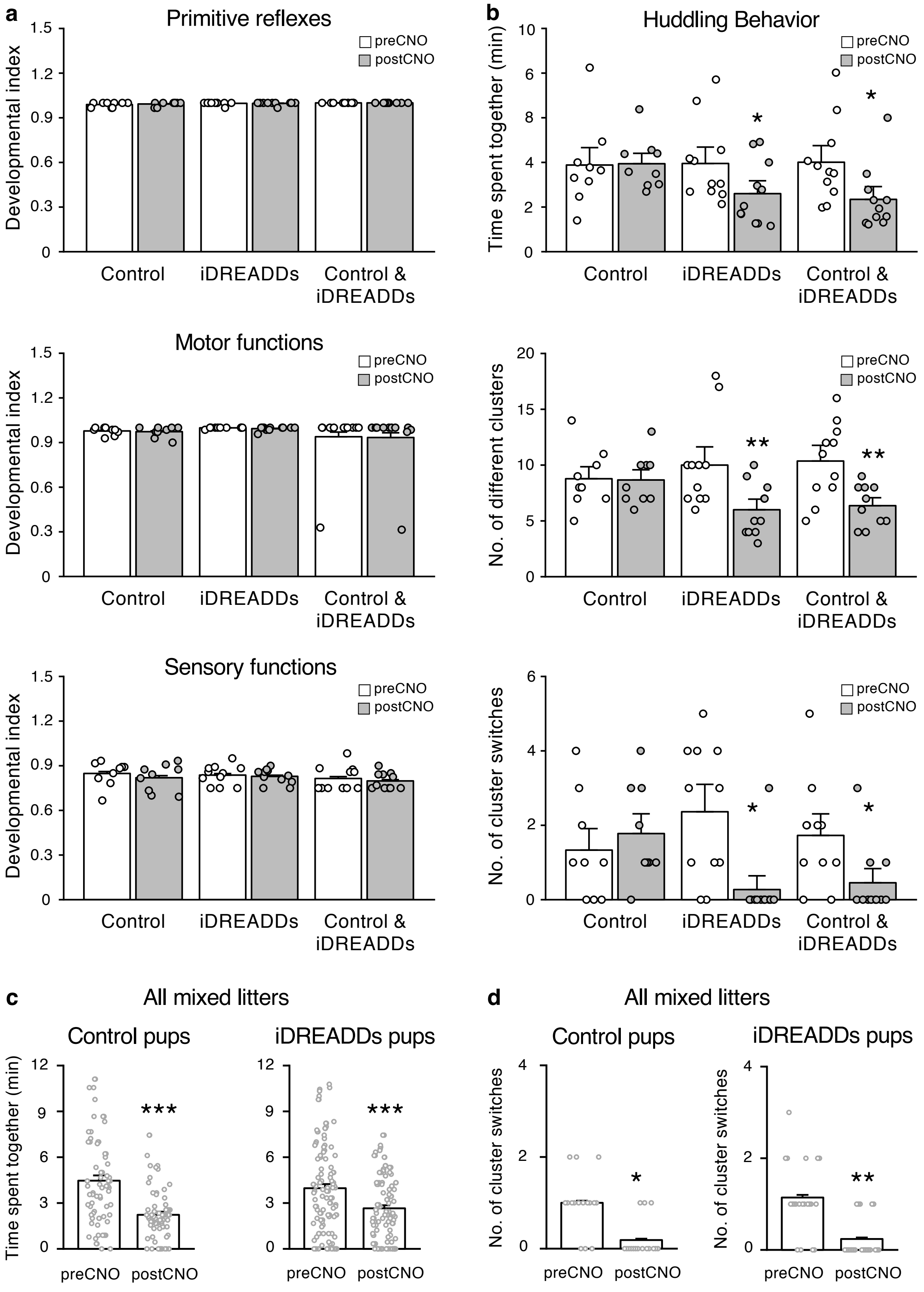
a
Primitive reflexes
Motor functions
Sensory functions
Developmental index
preCNO
postCNO
Control
iDREADDs
Control & iDREADDs
b
Huddling Behavior
Time spent together (min)
No. of different clusters
No. of cluster switches
c
All mixed litters
Control pups
iDREADDs pups
d
All mixed litters
Control pups
iDREADDs pups

Supplementary Figure 6. Huddling behavior depends on the activity of the somatosensory cortex. **a** Mean Developmental index ± SEM for primitive reflexes (top), motor functions (middle) and sensory functions (bottom) of the same sets of animals as examined in Fig. 3f, h and l before (white) and after CNO administration (gray). The interaction 'IUE group x treatment' was not significant in all the three behavioral classes: (Primitive reflexes: $F_{(2,28)}=1.24$, $P>0.05$; Motor functions: $F_{(2,28)}=0.005$, $P>0.05$; Sensory functions $F_{(2,28)}=0.72$, $P>0.05$, Supplementary Table 4). **b** Parameters describing the huddling behavior at P9 before and after CNO administration in the same sets of animals as examined in Figs 3g, i and m (top: Time spent together; middle: No. of different clusters; bottom: No. of cluster switches). Two-way ANOVA followed by pairwise *t*-test comparisons with Holm's correction: Time spent together ('IUE group x treatment' interaction: $F_{(2,28)}=3.18$, $P<0.05$); No. of different clusters ('IUE group x treatment' interaction: $F_{(2,28)}=6.87$, $P<0.01$); No. of cluster switches ('IUE group x treatment' interaction: $F_{(2,28)}=4.88$, $P<0.05$). **c** Average Time spent together ± SEM and data points for control-vector (Control, GFP) and iDREADD-vector (iDREADDs) electroporated individual pups from litters where both Control and iDREADDs pups were present, analyzed before and 30 minutes after CNO injection (Control: $N = 71$, two-sample paired *t*-test, t=6.2(70); iDREADDs: $N = 117$, Wilcoxon signed-rank test, W=4,700). The data set is the same as in Fig. 3. **d** Same as (**c**) but for No. of cluster switches (Control: $N = 16$; iDREADDs: $N = 21$; Wilcoxon signed-rank test, W=120 and 180 respectively); animals not performing any cluster switching were not considered for analysis. *$P<0.05$ and **$P<0.01$, ***$P<0.001$.

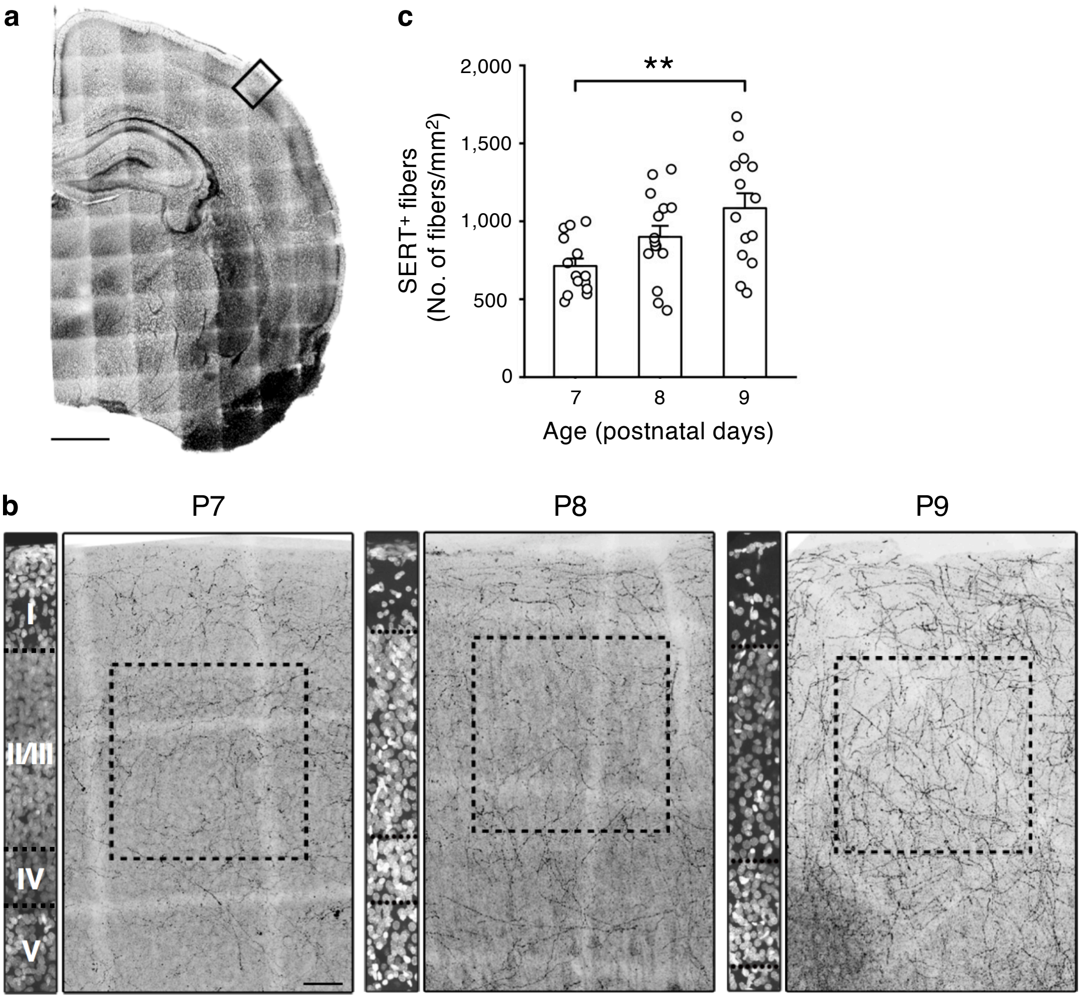

Supplementary Figure 7. SERT-positive processes massively invade the supragranular layers of the developing neocortex after P7. **a** Confocal images of SERT immunostaining in a brain slice at the level of the somatosensory cortex of a P7 rat. Scale bar: 1 mm. **b** Higher magnifications of regions as highlighted in (**a**). Dashed squares exemplify typical areas employed to quantify the number of SERT-positive fibers as in (**c**). Scale bar: 50 µm. Slices were counterstained with DAPI (left strips) for visualization of cortical layers necessary for drawing the squared regions of interest. **c** Average number ± SEM and single animal cases (average of two hemispheres of at least 2 slices/animal) of SERT-positive processes. The SERT-positive fibers of the supragranular layers increased significantly between P7 and P9 (N = 43 animals, Kruskal-Wallis test, $\chi^2_{(2)}$=8.6; Dunn's *post hoc* test with Holm's correction, **P<0.01).

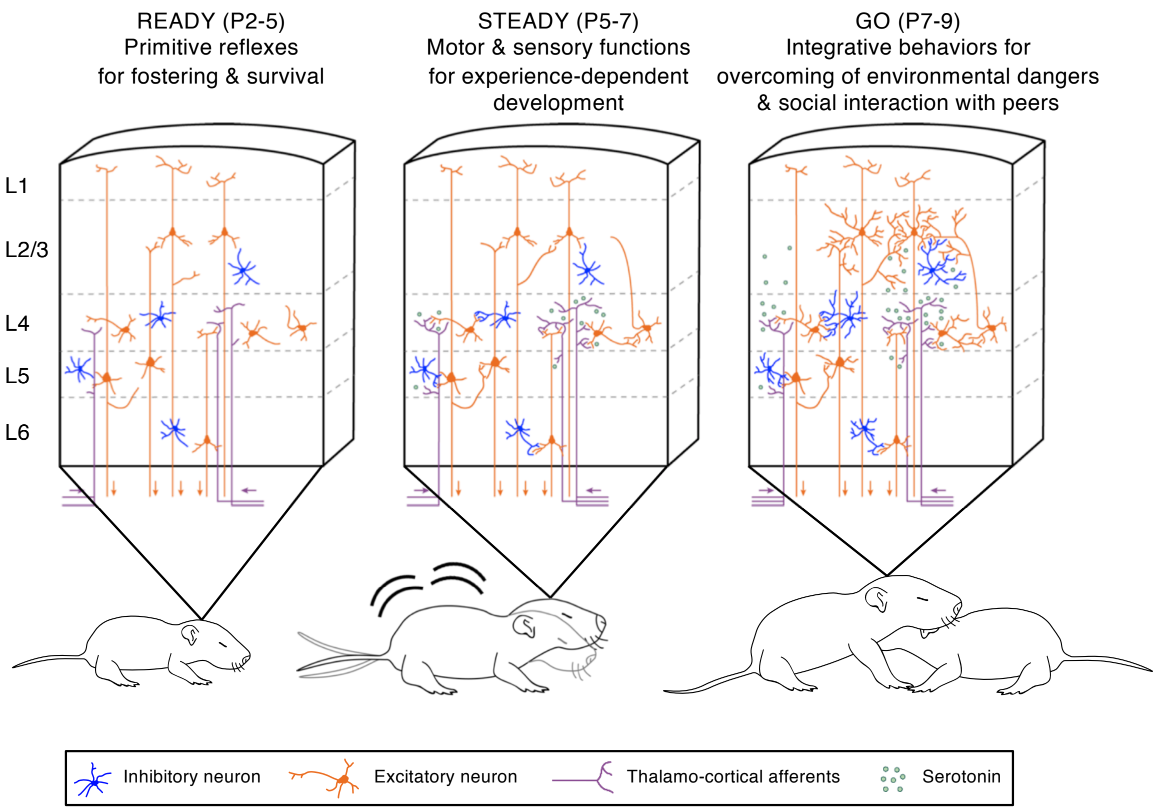

Supplementary Figure 8. Proposed model of neocortical development. Cartoon exemplifying the 'ready', 'steady', 'go' model for neocortical development. Migration of excitatory (orange) and inhibitory (blue) neurons in different cortical layers is completed by P5; the cortex enters a 'ready' state; pups have only primitive reflexes necessary for fostering. Then, thalamocortical axons (violet), which were already in place, start becoming branched and synaptically viable (including release of serotonin, green dots) around P7; synaptic transmission develops in the infragranular layers and the cortex enters a 'steady' state; pups acquire motor and sensory functions that are important for experience-dependent development, but also put the animals at risk of predation. Finally, at P9, synaptic transmission rapidly and sharply increases in the supragranular layers aided by an upsurge of 5-HT signaling and the cortex quickly enters in a safer 'go' state; pups are now able to integrate sensory and motor behaviors, enabling the animal to make the best of positive interaction with peers, while overcoming the dangers from the environment.

**Primitive reflexes**
• Suckling reflex (SUCR)
• Eye blink reflex (ER)
• Toe pinch reflex (TPR)
**Motor functions**
• Cliff avoidance (CA)
• Contact righting reflex (CRR)
• Gait (G)
• Trunk curl (TC)
• Grip strength (GS)
• Wire maneuver (WM)
• Negative geotaxis (NG)
**Sensory functions**
• Inner ear reflex (IER)
• Outer ear reflex (OER)
• Body position (BP)
• Spontaneous activity (SA)
• Transfer arousal (TA)
• Touch escape (TE)
• Positional passivity neck, tail and supine (PPNeck, PPTail, PPSupine)
• Limb grasping (LG)
• Whisker reflex (WR)

Supplementary Table 1. List of the different behaviors scored for the SHIRPA test. All the behaviors were divided into three different behavioral classes.

Name of the huddling parameter	Description	Individual behavior/ group behavior
Time spent together	The average time, expressed in minutes, that each pup spends forming a cluster with every other littermate during the entire huddling session (10 minutes). The individual measurements of all pups belonging to the same litter are then averaged to obtain a value representative of the corresponding litter.	Individual
Number of different clusters	For each litter, the number of clusters formed by unique combinations of different pups computed over the whole huddling session.	Group
Number of cluster switches	Number of times a pup switched clusters in the whole 10-minute recording period. A cluster was considered switched when this action occurred between two consecutive sampling intervals of 30 seconds each. For each litter, the sum of all the clusters formed by the pups during the huddling session was calculated.	Individual
Total space occupied	A grid formed by squares with an area of 18 cm^2 was created over the image on all the frames. The total space occupied was then defined as the number of different squares visited by each pup over all frames during the entire huddling session.	Individual

Supplementary Table 2. Huddling behavior parameters. Description of the different behaviors classified to score huddling (‘Time spent together’, ‘Number of different clusters’, ‘Number of cluster switches’) and motor ability (‘Total space occupied’).

Figure	Group	Statistical test	Test statistic	Degree of freedom
**3d**	Control	Wilcoxon signed-rank	W=18	
**3d**	iDREADDs	Wilcoxon signed-rank	W=18	
**3e**	Control	Paired *t*-test	t=-0.06	4
**3e**	iDREADDs	Paired *t*-test	t=6	4
**3f**	Primitive reflexes	Wilcoxon signed-rank	W=0	
**3f**	Motor functions	Wilcoxon signed-rank	W=5	
**3f**	Sensory functions	Paired *t*-test	t=1.7	8
**3g**	Time spent together	Paired *t*-test	t=-0.14	8
**3g**	No. of different clusters	Wilcoxon signed-rank	W=12	
**3g**	No. of cluster switches	Paired *t*-test	t=-0.71	8
**3h**	Primitive reflexes	#	#	#
**3h**	Motor functions	Wilcoxon signed-rank	W=6	
**3h**	Sensory functions	Wilcoxon signed-rank	W=35	
**3i**	Time spent together	Paired *t*-test	t=2.7	10
**3i**	No. of different clusters	Paired *t*-test	t=4.3	10
**3i**	No. of cluster switches	Wilcoxon signed-rank	W=36	
**3l**	Primitive reflexes	#	#	#
**3l**	Motor functions	Wilcoxon signed-rank	W=6	
**3l**	Sensory functions	Paired *t*-test	t=1.9	10
**3m**	Time spent together	Paired *t*-test	t=3.1	10
**3m**	No. of different clusters	Paired *t*-test	t=4.3	10
**3m**	No. of cluster switches	Paired *t*-test	t=2.5	10

Supplementary Table 3. Detailed statistical information for Figure 3. Name of statistical test, test statistic and degrees of freedom for all the experiments shown in Figure 3. Missing values (#) were due to the fact that all the values were identical.

**Group**	**Primitive reflexes**		**Motor functions**		**Sensory functions**	
	preCNO	postCNO	preCNO	postCNO	preCNO	postCNO
**Control**	0.99±0.00	0.99±0.00	0.98±0.00	0.98±0.01	0.85±0.01	0.82±0.01
**iDREADDs**	0.99±0.00	0.99±0.00	0.99±0.00	0.99±0.00	0.84±0.01	0.83±0.01
**Control & DREADDs mixed**	1.00±0.00	1.00±0.00	0.94±0.03	0.94±0.03	0.81±0.01	0.80±0.01

Supplementary Table 4. Descriptive statistics for Developmental index classes in Control, iDREADDs and Control & iDREADDs mixed litters. Means ± SEM of the Developmental index for the three different behavioral classes before and after CNO injection in Control, iDREADDs and Control & iDREADDs mixed litters (see Fig. 3f, h, l; Supplementary Fig. 3c and 6a).
