# Supplementary material for: The development of synaptic transmission is time-locked to early social behaviors in rats
Source: Nat Commun. 2019 Mar 13;10:1195. doi: 10.1038/s41467-019-09156-3 (PMC6416358; doi:10.1038/s41467-019-09156-3)
Supplement: Supplementary file 2 — Description of Additional Supplementary Files [file 41467_2019_9156_MOESM2_ESM.docx]

Description of Additional Supplementary Files

**Supplementary Movie 1:** Example of the formation of a huddling cluster in a P9 litter: pup #1 makes active huddling with pup #9.

**Supplementary Movie 2:** Example of a cluster switch recorded in a P9 litter: pup #4 switches from the cluster formed by pups #4,5,8 to the one formed by pups #3,9.
